# Aggravated renal fibrosis is positively associated with the activation of HMGB1-TLR2/4 signaling in STZ-induced diabetic mice

**DOI:** 10.1515/biol-2022-0506

**Published:** 2022-11-11

**Authors:** Yan Yuan, Yuanxia Liu, Mengyao Sun, Huijing Ye, Yuchen Feng, Zhenzhen Liu, Lingyu Pan, Hongbo Weng

**Affiliations:** Department of Pharmacology, School of Pharmacy, Fudan University, 3728 Jinke Road, Pudong District, Shanghai, 201203, China; Department of Pathology, Shanghai Municipal Hospital of Traditional Chinese Medicine, Shanghai University of Traditional Chinese Medicine, 274 Zhijiang Middle Road, Jing’an District, Shanghai, 200071, China

**Keywords:** diabetic kidney disease, high-mobility group box, Toll-like receptors, fibrosis, inflammation

## Abstract

Diabetic kidney dysfunction is closely associated with renal fibrosis. Although the suppression of fibrosis is crucial to attenuate kidney damage, the underlying mechanisms remain poorly understood. In this study, renal injury in diabetic mice was induced by the intraperitoneal injection of streptozotocin (100 or 150 mg/kg) for 2 consecutive days. In the model mice, remarkable renal injury was observed, manifested by albuminuria, swelling of kidneys, and histopathological characteristics. The renal fibrosis was obviously displayed with high-intensity staining of fibrin, type IV collagen (Col IV), and fibronectin. The levels of Col IV and transforming growth factor-β1 were significantly increased in diabetic mice kidneys. The aggravated fibrotic process was associated with the overexpression of HMGB1, TLR2/4, and p-NF-κB. Furthermore, a high expression of F4/80 and CD14 indicated that macrophage infiltration was involved in perpetuating inflammation and subsequent fibrosis in the kidneys of diabetic mice. The results demonstrate that the severity of renal fibrosis is positively associated with the activation of HMGB1/TLR2/4 signaling in diabetes.

## 1 Introduction

Diabetic kidney disease (DKD), one of the serious complications of diabetes, is the major cause of end-stage renal diseases [1,2,3]. In recent years, the worldwide prevalence of DKD has increased [4]. However, the knowledge gap of the pathogenesis of DKD leads to a lack of effective and safe therapeutics. At present, the treatment options available are almost suboptimal [4,5]. Therefore, an improved understanding of the pathogenesis of DKD is required for developing therapeutic strategies in clinical practice [6,7].

Kidney damage in diabetes is characterized by activated myofibroblasts, accumulation of fibrillary collagens, and inflammatory cells [8]. It has been reported that the sustained inflammation involved in DKD accelerates kidney injury, resulting in renal fibrosis [9,10]. As the late inflammatory factor, high-mobility group box (HMGB)-1 has been confirmed to be an important contributor to the maintenance of maintaining renal inflammation in DKD.

Under hyperglycemia and hyperlipidemia, HMGB1 is released from the cell nucleus and interacts with its receptors TLR2 and TLR4 on tubular cells, triggering an inflammatory response by activating the NF-κB signaling pathway to induce the production of proinflammatory cytokines [11]. Consequently, a high level of proinflammatory cytokines facilitates macrophage recruitment into kidney tissue, resulting in a self-perpetuating cycle of renal inflammation [12]. Meanwhile, several proinflammatory cytokines, including IL-1β, TNF-α, and IFN-γ, further stimulate macrophages to actively secrete HMGB1, which worsens the sustained inflammation of the kidney via positive feedback [13,14,15]. Therefore, the upregulation of HMGB1 gives rise to the amplification of inflammation and subsequent worsened kidney injury. In addition, HMGB1 exerts itself to facilitate collagen synthesis and accumulation [12]. Although the activation of HMGB-mediated TLR2/4 signaling has been analyzed in several mouse models of DKD, the correlation between HMGB1 activity and the progression of renal fibrosis is not well known [15,16,17].

In the present study, we refined the streptozotocin (STZ)-induced diabetic mouse model by administering different frequencies and doses. The model mice showed the remarkable kidney damage and renal fibrosis, especially at a higher dose of STZ. This aggravated fibrosis is associated with activated and augmented HMGB1 signaling. We conclude that the HMGB1/TLR2/4 pathway is involved in the regulation of fibrotic processes in the kidneys of STZ-induced diabetic mice, displaying a positive correlation.

## 2 Materials and methods

### 2.1 Establishment of the DKD mouse model

Male C57BL/6 mice (8 weeks old) were purchased from Slaccas-Shanghai Lab Animal Ltd. (SPF II Certificate; No. SCXK2012-0005) and kept under specific pathogen-free, normal housing conditions with constant humidity and temperature in a 12 h light and dark cycle.

After 1 week of being adapted to the new environment, mice were fasted but had free access to water overnight and subsequently treated with fresh STZ solution (Sigma-Aldrich, dissolved in 0.1 mmol/L sodium citrate buffer) by intraperitoneal injection in two doses (100 and 150 mg/kg) for 2 consecutive days (Days 0 and 1; *n* = 6 mice per group). On Day 8, blood glucose levels were monitored using a glucose meter (Abbott). Mice with blood glucose levels higher than 16.8 mmol/L were defined as hyperglycemic and used for experiments [18]. On Days 29 and 64, blood glucose levels were measured using assay kits following the manufacturers’ instructions (Fenghui, Shanghai, China). The survival status of the animals in each group was assessed during the whole experiment. At the end of the experiment (Day 64), serum and the kidneys of mice were collected for further assessment. All operations were performed anesthesia under 10% ethyl carbamate (intraperitoneal injection, 0.10 mL/10 g), and all efforts were taken to minimize the extent and the duration of the suffering. When euthanized in this experiment, mice were anesthetized with 10% ethyl carbamate followed by cervical dislocation.


**Ethical approval:** The research related to animal use has been complied with all the relevant national regulations and institutional policies for the care and use of animals, including the Guide for the Care and Use of Laboratory Animals of the National Institutes of Health. All the protocols in this experiment involving animals were approved by the Animal Ethical Committee of School of Pharmacy, Fudan University (approval number 2021-05-YL-WHB-78).

### 2.2 Biochemical assays

Urine was collected for 5 h via a device in a metabolism cage on Days 32 and 50. The concentration of urinary albumin was tested using an enzyme-linked immunosorbent assay (ELISA) kit, and the urinary albumin excretion rate (UAER) was calculated, indicating the total excretion of urinary albumin in 5 h.

On Day 64, blood was collected via the orbital sinus (under anesthesia with 10% ethyl carbamate, intraperitoneal injection, 0.10 mL/10 g) and centrifuged to obtain serum. High-density lipoprotein cholesterol (HDL-C), low-density lipoprotein cholesterol (LDL-C), serum creatinine, and blood urine nitrogen (BUN) were measured using assay kits (Fenghui, Shanghai, China).

### 2.3 Determination of inflammatory and fibrotic cytokines

The left kidney was weighed to calculate the kidney index (kidney weight/body weight, mg/g) and then homogenized in RIPA lysis reagent (Beyotime, Shanghai, China). The levels of TNF-α, IL-6, COI IV, and transforming growth factor (TGF)-β1 in the renal homogenates were measured using ELISA kits (Boatman, Shanghai, China).

### 2.4 Histopathological examination

On Day 64, the pancreas and the right kidney were collected, fixed in 10% formaldehyde, and embedded in paraffin wax. The specimens were processed using a microtome to obtain 4-μm-thick tissue sections. The tissue sections were stained with hematoxylin and eosin (H&E). Kidney sections were also processed for Periodic acid–Schiff (PAS) and Masson’s trichrome (Masson) assays.

### 2.5 Immunohistochemistry examination

The expression levels of HMGB1, TLR2, TLR4, F4/80, CD14, collagen IV (Col IV), and fibronectin (FN) in the kidney were examined using immunohistochemistry. The sections were dewaxed, rehydrated, and equilibrated in phosphate-buffered saline (pH 7.4). After quenching endogenous peroxidase activity with 3% H_2_O_2_ and blocking with 5% bovine albumin, the sections were incubated with antibodies recognizing HMGB1 (1:400, Proteintech), TLR2 (1:500, Arigo), TLR4 (1:400, Abcam), F4/80 (1:300, Servicebio), CD14 (1:500, Servicebio), Col IV (1:250, Abcam), and FN (1:250, Abcam) overnight. After incubation with a secondary antibody conjugated with horseshoe peroxidase [HMGB1, TLR2, TLR4, Col IV, and FN: horseradish peroxidase (HRP) 1:1,000, Bioworld; F4/80 and CD14: HRP 1:200, Servicebio] for 1 h at 37°C, signals were detected using the HRP substrate diaminobenzidine. The sections were lightly counterstained with hematoxylin and examined under a light microscope (Leica Inc., Switzerland).

### 2.6 Western blotting assay

Kidney specimens were homogenized in RIPA lysis reagent and centrifuged to collect the supernatant. The supernatant was mixed with the same volume of 2× SDS loading buffer. Total protein was measured by the Bradford assay (Beyotime, Shanghai, China).

Proteins in the samples were resolved in a 10% SDS polyacrylamide gel by electrophoresis and blotted onto polyvinylidene difluoride immobilon membranes (Millipore, Bedford, MA, USA). The membranes were blocked with 5% skim milk (in tris-buffered saline with Tween-20 buffer) for 1 h at room temperature and then probed overnight at 4°C with antibodies against HMGB1, p-NF-κB, FN, PCNA, and β-actin (all diluted at 1:1,000), followed by incubation with HRP-conjugated IgG (1:2,000, Beyotime, Shanghai, China) for 2 h at room temperature. Signals were detected by enhanced electrochemiluminescence reagent and captured with a camera-based imaging system (BIO-RAD, Santa Clara, CA, USA).

### 2.7 Statistical analysis

The data were expressed as mean ± SD. Differences between groups were analyzed by one-way analysis of variance (ANOVA), and *post hoc* comparisons of Fisher’s PLSD were performed. A *P*-value of <0.05 was considered significant.

## 3 Results

### 3.1 Characteristics of STZ-induced diabetic mice

**Figure 1:**
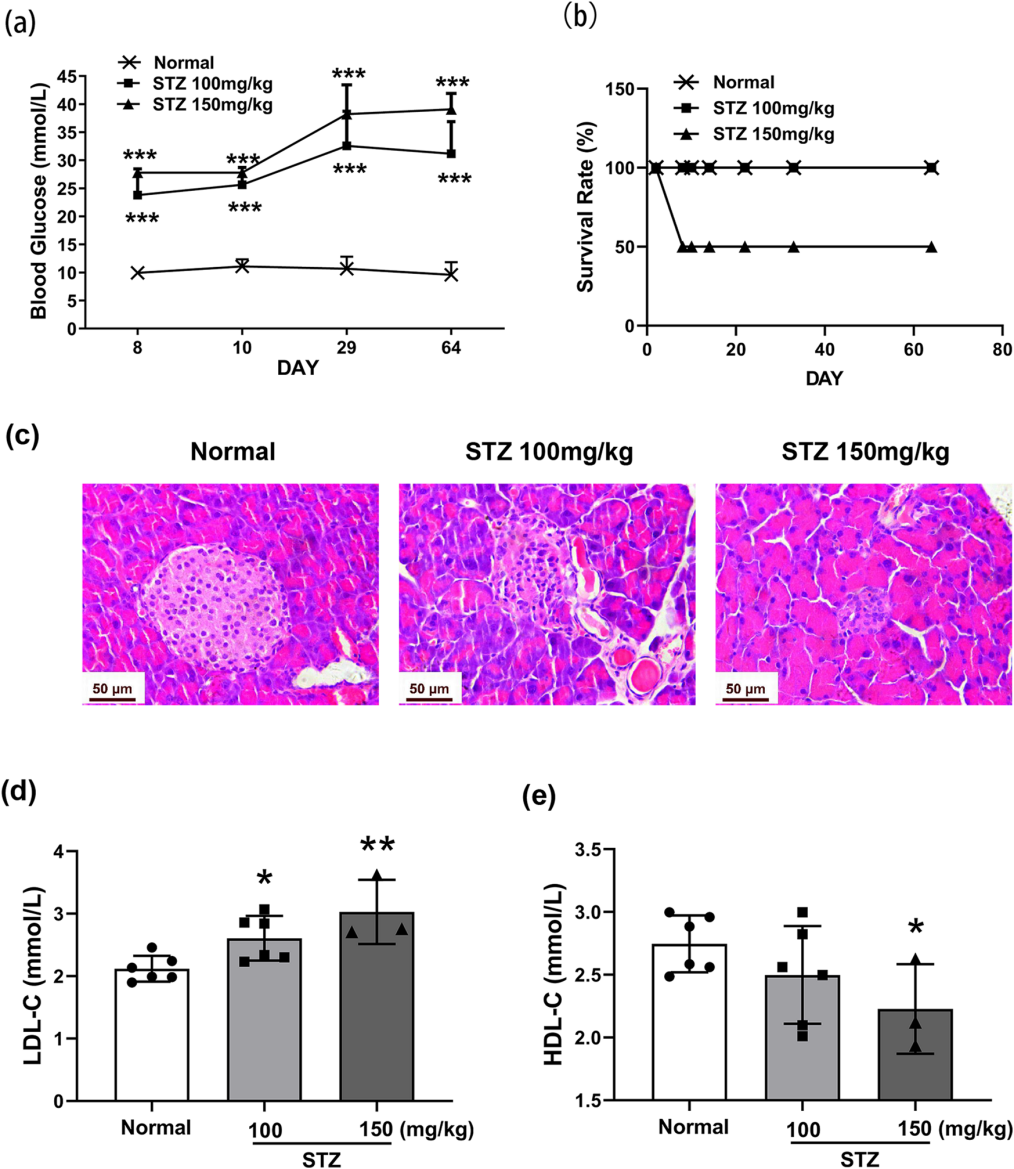
Characteristics of metabolism and histopathology in STZ-induced diabetic mice. Mice were treated with STZ at 100 or 150 mg/kg by intraperitoneal injection. The pancreas was dissected on Day 64. The blood glucose levels (a) and survival rate (b). Histopathology of the pancreas detected by H&E, photographed at 400× magnification (c). The levels of serum LDL-C (d) and HDL-C (e) in STZ-induced mice, *n* = 6. Data are expressed as the means ± SD. *P < 0.05, **P < 0.01, ***P < 0.001, vs the normal group, tested by one-way ANOVA and Fisher’s PLSD.

Hyperglycemia was achieved (≥16.8 mmol/L) on Day 8 and sustained thereafter in the STZ-treated groups, as compared with the normal group (*P* < 0.001; Figure 1a). Mice with blood glucose levels higher than 27 mmol/L and weight lower than 16 g were euthanized by excessive anesthesia to minimize suffering. Survival rates up to Day 64 after STZ treatment were 100 and 50% in the 100 and 150 mg/kg groups, respectively (Figure 1b).

At the histological level, the Langerhans’ islets of the pancreas exhibited normal circular morphology with a healthy cell lining in the normal mice (Figure 1c). The exocrine glands of the pancreas were well organized and hadnormal morphology. In contrast, in the 100 mg/kg STZ group, the islet cell mass appeared atrophied and disorganized. In the 150 mg/kg STZ group, the islets of Langerhans further shrank and were almost indiscernible (Figure 1c).

Hyperlipidemia is an indicator of severe diabetes. Compared with the normal group, the levels of serum LDL-C in the diabetic groups (treated with 100 or 150 mg/kg STZ) were significantly higher (*P* < 0.05, *P* < 0.01, respectively) (Figure 1d), while the levels of serum HDL-C (150 mg/kg STZ) were significantly lower (*P* < 0.05, Figure 1e).

### 3.2 Renal injuries in STZ-induced diabetic mice

**Figure 2:**
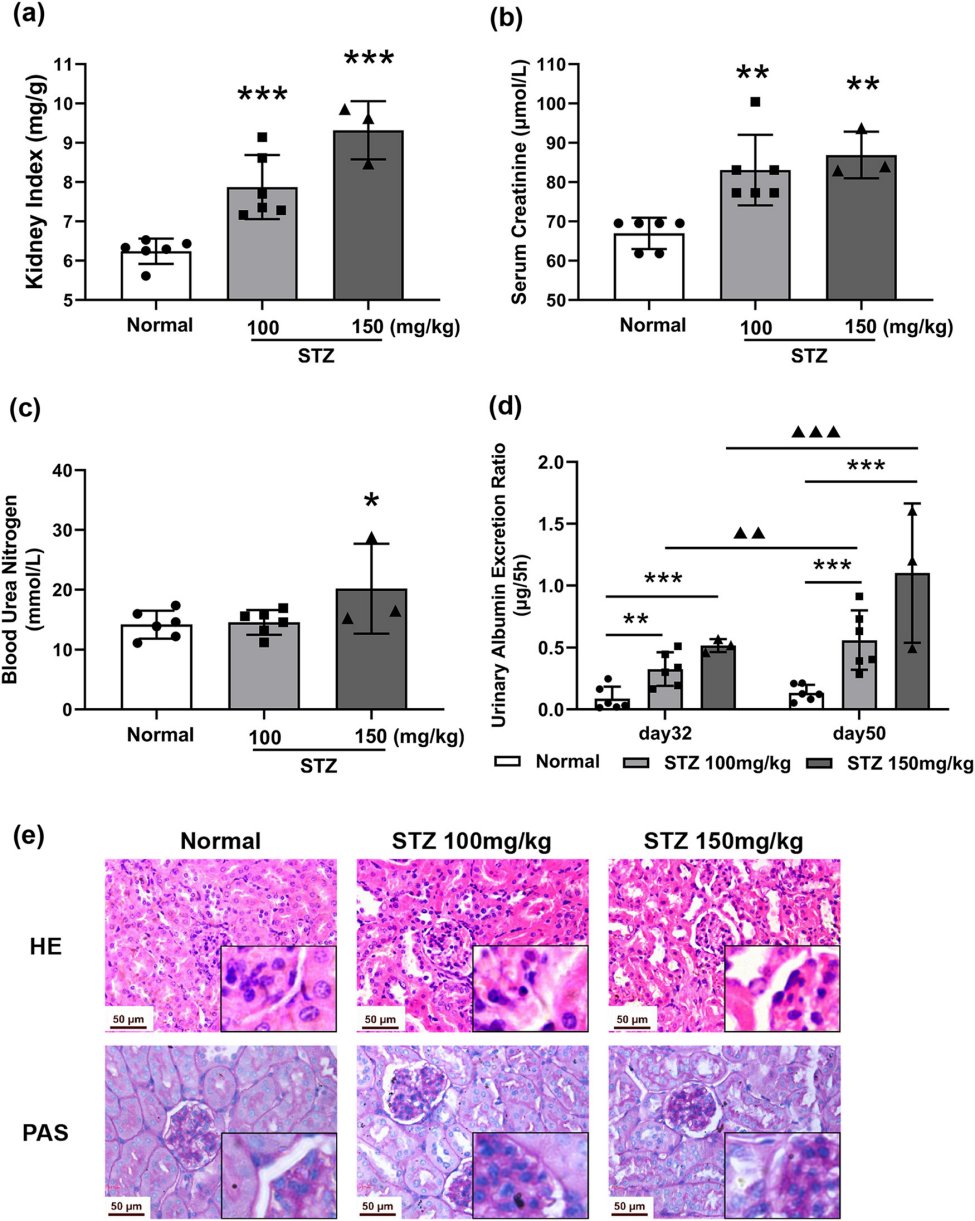
Kidney damaged in STZ-induced diabetic mice. Mice were treated with STZ at 100 or 150 mg/kg by intraperitoneal injection. Urine was collected for 5 h via a device in a metabolism cage at Days 32 and 50. The kidney was dissected on Day 64. The kidney index (a), serum creatinine (b), blood urea nitrogen level (c), urinary albumin excretion rate (UAER) (d), and renal histological changes (e) H&E, PAS, photographed at 400× magnification. *n* = 6, Data expressed as the mean ± SD. *P < 0.05, **P < 0.01, ***P < 0.001 vs the normal group, tested by one-way ANOVA and Fisher’s PLSD.

The kidney index (*P* < 0.001, Figure 2a) and serum creatinine level (*P* < 0.05, Figure 2b) were significantly increased in the diabetic groups, compared with those of the normal group. There was no difference in the levels of BUN (Figure 2c).

The levels of UAER were significantly elevated in the diabetic groups, compared with those of the normal group, and presented a progressive increase during the experiment (*P* < 0.001, Figure 2d).

H&E examination indicated pathological characteristics in the kidneys of DKD mice such as expansion of the glomerular mesangial region (Figure 2e). The PAS assay showed intensive deposition of saccharides in the glomerular mesangial region in the diabetic mice, indicating the proliferation of the extracellular matrix (Figure 2e).

### 3.3 Renal fibrosis in STZ-induced diabetic mice

**Figure 3:**
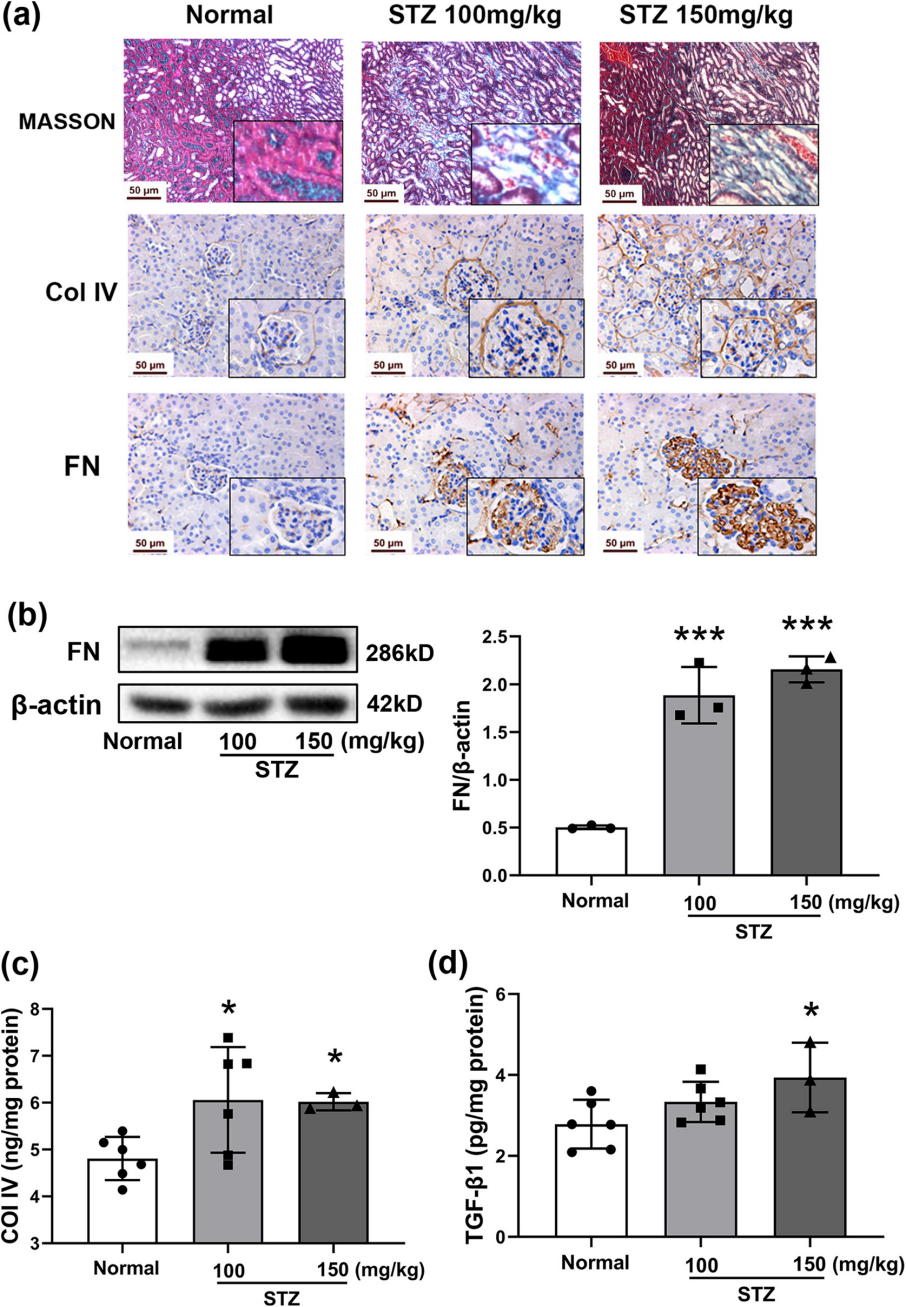
Severity of renal fibrosis in STZ-induced diabetic mice. Mice were treated with STZ at 100 or 150 mg/kg by intraperitoneal injection. The kidney was dissected on Day 64. Histological assays of the kidney (Masson’s trichome), immunohistochemical staining of Col IV and FN in the kidney (a), photographed at 400× magnification. Protein expression of FN in the kidney by Western blot (b), *n* = 3. β-Actin was used as an internal control. The levels of Col IV (c) and TGF-β1 (d) in renal tissues tested by ELISA. Data are expressed as the mean ± SD. *P < 0.05, ***P < 0.001 vs the normal group, tested by one-way ANOVA and Fisher’s PLSD.

Masson’s trichrome assay showed vacuolation of the tubular cells and heavy deposition of fibroblasts and fibrillary collagens in the tubular interstitium (Figure 3a). Col IV and FN, indicators of fibrosis, were highly accumulated in the kidneys of diabetic mice as assessed by immunohistochemical staining. Heavy Col IV deposition was seen in the renal tubule interstitial and basement membranes, while FN was highly present in the mesangial glomerulus and renal tubule interstitial (Figure 3a). The data from Western blot assays confirmed the upregulation of FN in the kidneys of model mice (*P* < 0.01; Figure 3b). Consistently, the levels of Col IV and FN were significantly elevated in the kidney homogenates of diabetic mice, compared with those of the normal group (*P* < 0.05; Figure 3b and c). TGF-β1 is well recognized as a risk factor that promotes fibrosis. In this study, the production of TGF-β1 displayed a significant increase in the renal tissues of diabetic mice (Figure 3d).

### 3.4 Renal inflammation in STZ-induced diabetic mice

**Figure 4:**
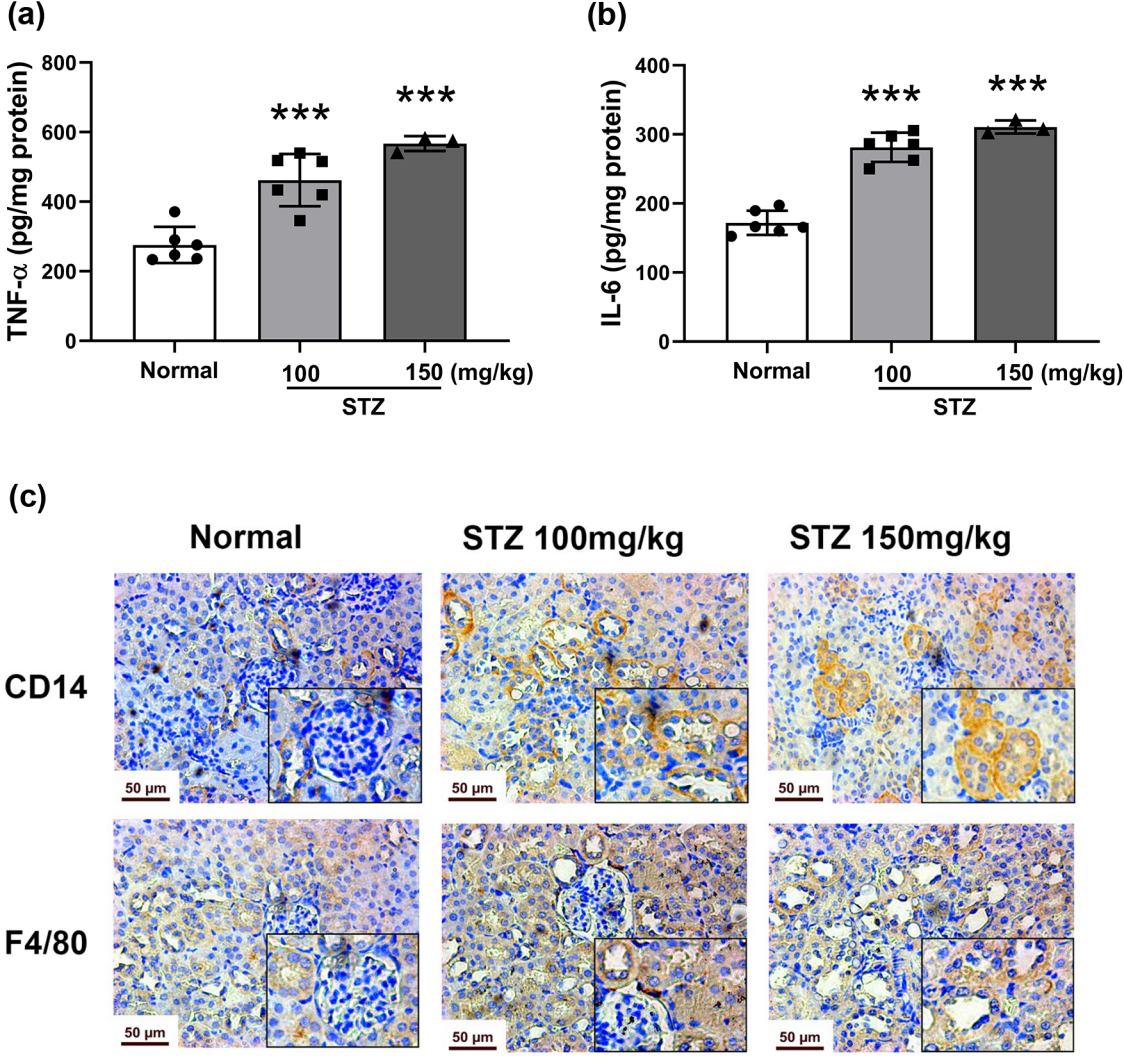
Level of inflammatory cytokines and macrophage infiltration in the kidneys of STZ-induced diabetic mice. Mice were treated with STZ at 100 or 150 mg/kg by intraperitoneal injection. The kidney was dissected on Day 64. The levels of the inflammatory cytokines TNF-α (a) and IL-6 (b) in renal tissues tested by ELISA, *n* = 6. Immunohistochemical staining (c) of CD14 and F4/80 in the kidneys of STZ-induced mice, photographed at 400× magnification, *n* = 3. Data are expressed as the mean ± SD. *P < 0.05, **P < 0.01, ***P < 0.001, vs the normal group, tested by one-way ANOVA and Fisher’s PLSD.

Inflammation is a key contributor to the development of the fibroblast activation process in the kidneys of diabetic mice. The inflammatory cytokines in the kidney homogenate were tested by ELISA. In the diabetic groups, the levels of TNF-α and IL-6 significantly ascended, compared with those in the normal group (*P* < 0.001; Figure 4a and b). To further evaluate the inflammatory condition, the macrophage infiltrate was determined by immunohistochemical assay. The results showed that F4/80 and CD14 were highly expressed and caused the inflammation infiltration in the kidneys of diabetic mice (Figure 4c), which contributed to amplification and perpetuation of inflammation.

### 3.5 Expression of HMGB1, TLR2/4, and p-NF-κB p65 in STZ-induced diabetic mice

**Figure 5:**
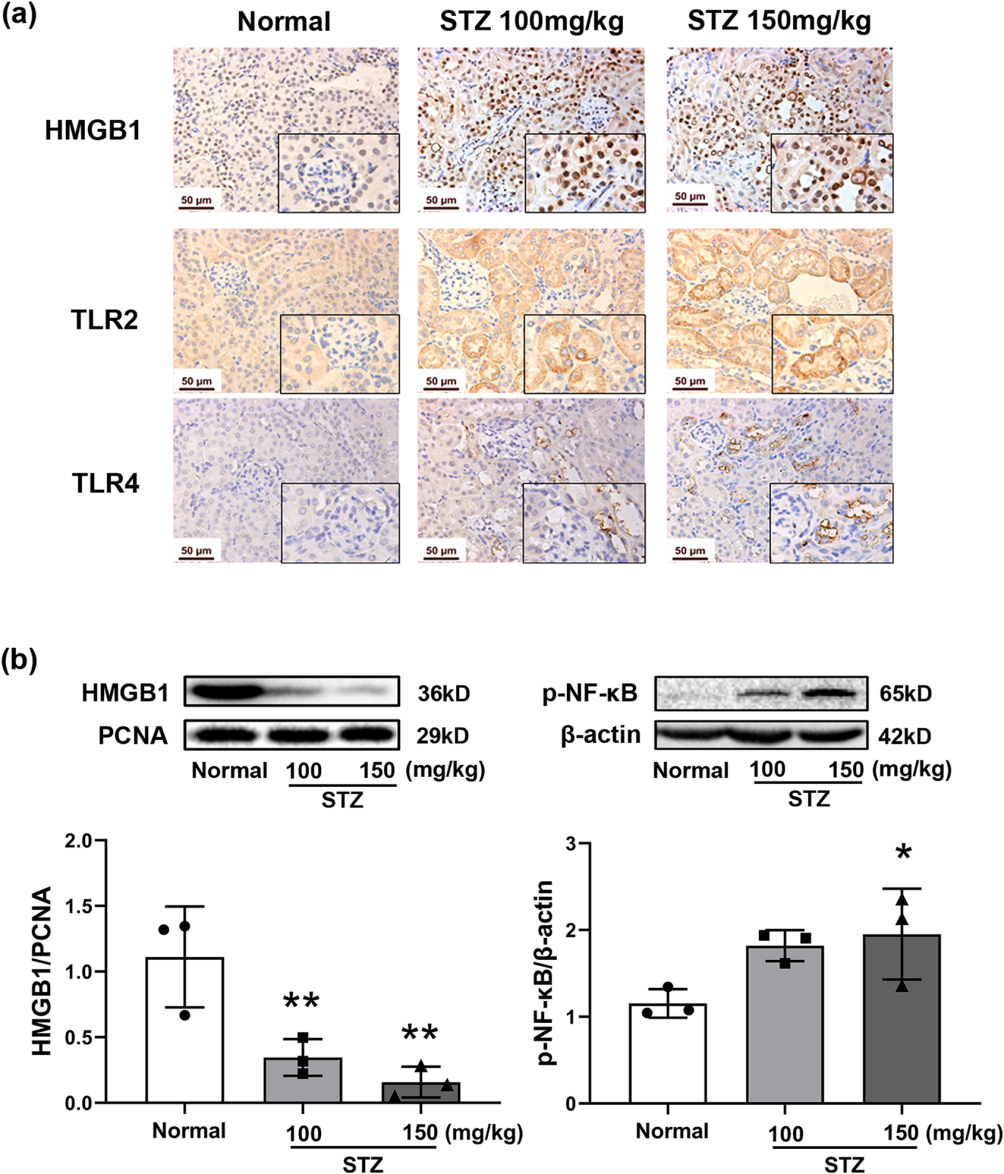
Activation of the HMGB1/TLR2/4 signaling pathway in the kidneys of STZ-induced diabetic mice. Mice were treated with STZ at 100 or 150 mg/kg by intraperitoneal injection. The kidney was dissected on Day 64. Immunohistochemical staining of HMGB1, TLR2, and TLR4 in the kidneys (a), photographed at 400× magnification. Protein expression of HMGB1 and p-NF-κB in the kidneys by Western blot (b), *n* = 3. PCNA and β-actin were used as internal controls. Data are expressed as the mean ± SD. *P < 0.05, **P < 0.01, ***P < 0.001, vs the normal group, tested by one-way ANOVA and Fisher’s PLSD.

As shown in Figure 5a, HMGB1, TLR2, and TLR4 were expressed at low levels in the normal mice but were notably upregulated in the kidneys of diabetic mice. Localization of HMGB1 was observed to be in the cytoplasm and extracellular space in the kidney of diabetic mice while was mainly in the nucleus in the normal group (Figure 5a). Western blot assays were consistent with the immunohistochemical results of the lower level of HMGB1 in the nucleus in the model mice (Figure 5b). The data suggested that the HMGB1 was not only highly expressed but also released into the cytoplasm and extracellular space, contributing to the activation of the downstream signaling pathway in the kidneys of diabetic mice.

NF-κB is an important transcription factor in TLR signaling that regulates the expression of numerous inflammatory genes [19]. As shown in Figure 5b, the level of p-NF-κB expression was obviously increased in the renal tissues of diabetic mice based on Western blotting. Cumulatively, the results indicated that the activation of HMGB1-TLR 2/4 signaling promoted renal fibrosis via NF-κB-mediated production of proinflammatory cytokines.

## 4 Discussion

This study demonstrates that the progression of renal fibrosis is related to the activation of HMGB1 in the STZ-induced diabetic mouse model. In this model, injection of 100 and 150 mg/kg STZ both successfully induced renal injuries and fibrosis. Considering the mortality of model mice, 100 mg/kg STZ is recommended to establish the DKD mouse model. By means of the model, our results demonstrate that the amplification of HMGB1 signaling is an important contributor to the worsened kidney damage.

The accumulation of fibrillary collagens and inflammatory cells is the obvious characteristics of DKD, resulting in renal failure [8]. Although the HMGB1 interaction with TLRs via stimulation of the NF-kB pathway has been analyzed in several mouse models of DKD, the correlation between HMGB1 level and the progression of renal fibrosis is not well known [15,16,17] which slows the process of research on targeting HMGB1 for DKD therapy. In the present study, we refined the STZ-induced diabetic mouse model by administering different frequencies and doses to observe the effect of HMGB1 on renal fibrosis in DKD mice at different time points. Increased levels of UAER, serum creatinine, and kidney index were observed in STZ-induced mice. The histological examination was consistent, including HE, PAS, and Masson assays. To evaluate the degree of renal fibrosis, the proteins and cytokines related to the fibrosis process were detected. The results showed that both Col IV and FN were highly expressed and accumulated in the kidneys of diabetic mice, and the levels of Col IV and TGF-β1 increased in kidney homogenate. Therefore, the model enabled us to investigate the key factor in the progression of renal fibrosis in diabetic mice, which is much more intuitive to observe the effect of HMGB1 on the progression of renal fibrosis in DKD.

The occurrence and development of kidney damage are a result of multiple factor interactions under hyperglycemic environments [1,20,21]. Accumulating evidence indicates that inflammation plays a critical role in the progression of DKD [1]. As pattern recognition receptors, TLRs can bind with their endogenous ligands and then activate downstream NF-κB signaling pathways to trigger an inflammatory response when subjected to noninfectious stimuli, such as hyperglycemia [22,23]. The elevated production of proinflammatory cytokines is suggested to play a critical profibrotic role in diabetes [24]. Our data demonstrated that the levels of TNF-α and IL-6 were increased in the kidney homogenate of DKD model mice, and the alteration was associated with the severity of renal fibrosis.

In general, elevated levels of proinflammatory cytokines facilitate macrophage recruitment into kidney tissue, manifesting inflammatory infiltration [25,26]. Subsequently, activated macrophages further release more cytokines, forming a positive feedback regulation and amplification of inflammatory signaling [8,27]. Therefore, macrophage infiltration is known to be an important contributor to the progression of renal fibrosis. Consistently, our results showed that the levels of F4/80 and CD14 were markedly increased in the kidneys of diabetic mice by immunohistochemical staining, indicating that the kidneys of our model were exposed to the persistent induction of worsened renal fibrosis.

HMGB1 is a nuclear DNA-binding protein that modulates chromatin accessibility [28]. However, it can translocate into the cytoplasm from the nucleus and be released in the necrotic and damaged cells or activated immune cells [29]. Extracellular HMGB1 promotes inflammation asone of the ligands of TLR2 and TLR4 by activating downstream NF-κB signaling pathways [12,29,30]. Our data showed that the expression and translocation of HMGB1 were elevated in the kidneys of our model mice, accompanied by an increase in TLR2 and TLR4 expression and phosphorylated NF-κB. Cumulatively, the results demonstrated that HMGB1 expression was well associated with STZ-induced renal fibrosis after 2 consecutive days of 100 mg/kg STZ injection intraperitoneally. The findings are beneficial to further explore the role of HMGB1 in anti-fibrosis therapeutic strategies of DKD. In addition, our study provides an available and stable animal model for DKD investigation.

In this study, although the results from the study confirm that the activation of HMGB1/TLR2/4 contributes to the renal fibrosis process, the effect of the suppression of HMGB1 expression remains unclear and future experiments are needed. Moreover, the interactions between HMGB1 and other receptors or pro-inflammatory cytokines are complicated and interesting; further study is needed to fully understand this complexity.

## 5 Conclusion

In conclusion, on 2 consecutive days in a 100 mg/kg STZ-induced renal fibrosis mouse model, our results demonstrate that aggravated renal fibrosis is positively associated with the activation of HMGB1/TLR2/4 signaling. This study provides insight into anti-inflammatory therapeutic strategies for attenuating renal fibrosis in diabetes.
